# A Cross-Sectional Study of Word Recognition and Cognitive Function in Older Adults with Hearing Loss Using a Standardized Neuropsychological Battery

**DOI:** 10.3390/jcm14227897

**Published:** 2025-11-07

**Authors:** Hyun Jin Lee, Tae Hoon Kong, Kyoung Ho Park

**Affiliations:** 1 Department of Otorhinolaryngology—Head and Neck Surgery, Incheon St. Mary’s Hospital, College of Medicine, The Catholic University of Korea, Seoul 21421, Republic of Korea; 2 Department of Otorhinolaryngology—Head and Neck Surgery, Yonsei University Wonju College of Medicine, 20 Ilsan-ro, Wonju 26426, Republic of Korea; 3 Department of Otolaryngology—Head and Neck Surgery, College of Medicine, The Catholic University of Korea, Seoul 06591, Republic of Korea

**Keywords:** hearing loss, speech discrimination score, K-MMSE, SNSB, cognitive impairment

## Abstract

**Objectives**: Dementia and hearing loss are prevalent conditions among older adults. This study aimed to determine the association between hearing loss and cognitive status using data from the Catholic Medical Center Clinical Data Warehouse (CMC–CDW). **Methods**: A retrospective review was conducted using the CMC–CDW. A total of 801 participants, aged 60 years and older, underwent bilateral speech audiometry and cognitive assessments using the Korea Mini-Mental State Examination (K–MMSE) and the Seoul Neuropsychological Screening Battery (SNSB). **Results**: The mean age of the participants was 77.1 ± 9.7 years, and the sex distribution was 313 males and 488 females. The mean speech recognition threshold was 39.6 ± 4.8 dB, and the average speech discrimination score was 74.3 ± 29.9%. The mean K–MMSE score was 25.1 ± 4.3. Cognitive status was categorized as normal (n = 205), mild cognitive impairment (n = 438), and dementia (n = 158). Logistic regression revealed that age, sex, and hearing loss were significantly associated with cognitive impairment (*p* < 0.05). **Conclusions**: These findings suggest that the association between hearing loss and cognitive impairment may be observed even at a mild stage of hearing loss, highlighting the need for early evaluation and management in older adults.

## 1. Introduction

Age-related hearing loss, or presbycusis, refers to the gradual, bilateral decline in hearing sensitivity that predominantly affects high-frequency sounds such as ringing phones or beeping microwaves. Aging has been consistently linked to both hearing loss and cognitive decline [1,2]. Word recognition ability is a critical indicator of functional hearing and plays a central role in everyday communication as well as cognitive health [3]. Impairments in word recognition may reflect not only peripheral auditory deficits but also diminished central auditory processing and weakened cognitive domains, including attention and working memory. Recent evidence demonstrates that both word recognition and cognitive function are key determinants of real-world functional limitations, influencing activities of daily living and social participation in older adults [3,4].

Globally, the prevalence of dementia among individuals aged ≥60 years is estimated at 5–7%, with rates doubling approximately every 20 years [5]. Simultaneously, over 35% of individuals in their 60s and more than 50% in their 70s experience hearing loss that disrupts daily functioning [6]. Longitudinal studies of community-dwelling older adults have demonstrated a 30–40% increased risk of accelerated cognitive decline associated with hearing loss [7].

Cognitive impairment encompasses a spectrum of conditions ranging from mild cognitive impairment to advanced dementia, while hearing loss varies in severity and substantially affects communication and quality of life [8,9]. Cognitive abilities influence speech recognition performance, and untreated hearing loss exacerbates cognitive dysfunction [10].

Speech intelligibility is commonly assessed through word recognition tests, which measure a listener’s ability to identify phonetically balanced words at their most comfortable listening level [11]. The highest score achieved, known as the phonetically balanced maximum (PBmax), reflects both peripheral and central auditory processing [12]. Considering these interrelationships, a comprehensive assessment combining both word recognition and neuropsychological testing provides valuable insight into the multidimensional impacts of age-related hearing loss—including its relationship with cognitive decline and broader functional limitations.

The Korean Mini-Mental State Examination (K-MMSE) is a widely used and validated screening tool that assesses global cognitive function, including orientation, attention, memory, language, and visuospatial abilities. Lower scores indicate greater cognitive impairment, and established age- and education-adjusted cut-off values improve diagnostic accuracy [13]. The Seoul Neuropsychological Screening Battery (SNSB) provides a comprehensive evaluation across five cognitive domains (attention, language, visuospatial function, memory, and executive function), allowing for detection of both global and domain-specific impairments [14]. Together, these complementary measures ensure robust, multidimensional assessment of cognitive status in older adults with hearing loss.

Recent research suggests that age-related hearing loss may accelerate cognitive decline through several mechanistic pathways, including increased cognitive load, brain atrophy, and chronic neuroinflammation. Structural and functional brain changes observed in hearing-impaired individuals provide biological plausibility for this association. Prolonged auditory deprivation may also limit cognitive and social stimulation, further contributing to decline. Based on these mechanisms, we hypothesize that hearing deficits, particularly those reflected in speech discrimination ability, are associated with cognitive impairment in older adults [4,15,16].

This study aimed to examine the association between cognitive function and speech discrimination ability in older adults with hearing loss using standardized neuropsychological assessments and speech audiometry.

## 2. Materials and Methods

### 2.1. Study Population

This retrospective, cross-sectional study investigated the association between hearing loss and cognitive function in older adults. Clinical and audiological data (January 2008 and December 2018) were extracted from the Catholic Medical Center Clinical Data Warehouse (CMC–CDW), which is a centralized repository of anonymized electronic medical records from Seoul St. Mary’s Hospital, a tertiary referral center affiliated with the Catholic University of Korea.

Participants were eligible if they were aged 60 years or older, had a diagnosis of sensorineural hearing loss (SNHL), and completed both the Korean Mini-Mental State Examination (K–MMSE) and the Seoul Neuropsychological Screening Battery (SNSB). Sociodemographic characteristics, including age, sex, and educational attainment, were systematically collected and included as covariates in the analyses.

**Figure 1:**
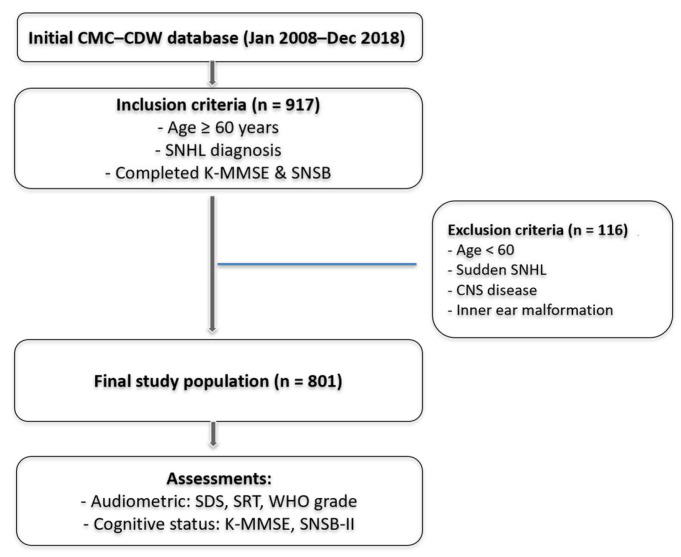
Flowchart illustrating the study design, including patient selection from the CMC–CDW database, inclusion and exclusion criteria, and the audiological and cognitive assessments conducted.

Exclusion criteria were as follows: (1) age <60 years; (2) sudden sensorineural hearing loss; (3) central nervous system diseases, including stroke or other cerebrovascular disorders; or (4) inner ear diseases or congenital malformations. A flowchart summarizing participant selection, exclusion criteria, and key assessments is provided in Figure 1.

### 2.2. Audiometric Measurements

Speech audiometry was performed in a sound-attenuated booth by a trained technician. To assess speech discrimination ability, word recognition tests were conducted to determine the speech discrimination score (SDS), representing the maximum percentage of correctly identified words at the most comfortable listening level. The test used 50 monosyllabic Korean words selected from a validated, standardized phonetically balanced list established in a prior study [17]. All speech audiometric evaluations were conducted using live voice by a single experienced female audiologist to ensure procedural consistency and minimize inter-rater variability. Masking noise was applied to the contralateral ear when necessary to prevent cross-hearing. Sound intensity was standardized using a calibrated microphone to maintain reliability.

Hearing status was classified based on the Speech Recognition Threshold (SRT), defined as the minimum sound intensity (dB HL) at which participants correctly repeated 50% of standard Korean bisyllabic words, presented via live voice. The SRT was measured using a validated list of 36 Korean spondee words and the modified ascending method, following Korean clinical audiology standards [18]. Hearing loss severity was categorized according to SRT-based WHO criteria: normal (<20 dB HL), mild (20–34 dB HL), moderate (35–49 dB HL), moderately severe (50–64 dB HL), severe (65–79 dB HL), and profound (≥80 dB HL).

### 2.3. Assessment of Cognitive Function

Cognitive function was evaluated using the K-MMSE and the Seoul Neuropsychological Screening Battery (SNSB-II). The K-MMSE is a 30-item test covering orientation (10), registration (3), attention/calculation (5), recall (3), language (8), and visuospatial construction (1), with cognitive impairment defined by age- and education-adjusted cut-offs [19].

The SNSB-II includes 29 subtests assessing five domains—attention, language and related functions, visuospatial function, memory, and executive function—classified using domain-specific Z-scores, which are calculated based on normative data; scores below −1 SD were categorized as ‘impaired’ for that domain [14]. Cognitive status was categorized as normal, mild cognitive impairment (MCI), mild dementia, and moderate to severe dementia, based on established K-MMSE and SNSB criteria.

### 2.4. Outcome Measures

The primary outcome variables were speech discrimination score (SDS), WHO hearing classification (grades 0–5), and cognitive status (normal cognition, mild cognitive impairment, or dementia). Domain-specific SNSB scores were also analyzed. For subgroup analysis, performed to strengthen interpretability, hearing loss was dichotomized into mild-to-moderate (26–60 dB HL) and severe-to-profound (>60 dB HL) categories, based on WHO classification. Cognitive status was further categorized as normal or impaired (including mild cognitive impairment and dementia). The Prevalence Ratios (PR) and 95% Confidence Intervals (CI) of cognitive impairment in each group were calculated using crude ratios. Categorical and continuous variable boundaries were based on WHO criteria and established literature. The primary variables analyzed were age, sex, education, speech recognition threshold (SRT), speech discrimination score (SDS), and cognitive status (normal, mild cognitive impairment, dementia). Hearing loss severity was classified using the World Health Organization (WHO) SRT-based criteria: normal (<20 dB HL), mild (20–34 dB HL), moderate (35–49 dB HL), moderately severe (50–64 dB HL), severe (65–79 dB HL), and profound (≥80 dB HL) [20]. All classification and modeling procedures were pre-specified and are described to ensure reproducibility.

### 2.5. Statistical Analysis

All statistical analyses were conducted using SAS version 9.4 (SAS Inc., Cary, NC, USA). Continuous variables were expressed as means with standard deviations, and categorical variables as frequencies and percentages. Group comparisons across hearing loss severity and cognitive status were performed using one-way ANOVA and chi-square tests. A two-tailed *p*-value < 0.05 was considered statistically significant. To evaluate the association between hearing loss and cognitive impairment, multivariate logistic regression was performed with cognitive impairment (normal vs. mild cognitive impairment [MCI] or greater) as the dependent variable. Independent variables included age, sex, speech recognition threshold (SRT), SDS, and WHO hearing classification. Model selection was guided by the Akaike Information Criterion (AIC) [20]. All analyses were pre-specified and followed the Strengthening the Reporting of Observational Studies in Epidemiology (STROBE) guidelines.

## 3. Results

A total of 801 participants aged 60 years or older were included in the analysis. The mean age was 77.1 ± 9.8 years, and 60.9% of the participants were female. The SDS was 73.4 ± 29.9% in the right ear and 71.2 ± 31.8% in the left ear. The average K-MMSE score was 25.1 ± 4.3. The Clinical Dementia Rating (CDR) distribution was 8.7% with CDR 0 (no impairment), 78.9% with CDR 0.5 (questionable), 9.0% with CDR 1.0 (mild), 3.2% with CDR 2.0 (moderate), and 0.1% with CDR 3.0 (severe). Based on cognitive status, 25.6% of participants were cognitively normal (n = 205), 54.7% had mild cognitive impairment (MCI, n = 438), 14.3% had mild dementia (n = 115), and 5.3% had moderate to severe dementia (n = 43) (Table 1).

### Group Comparisons by Hearing Loss Severity

When stratified by WHO hearing loss classification (based on the worse ear), significant group differences were observed in age, speech recognition thresholds (SRT), SDS, and cognitive performance (all *p* < 0.001). Participants with normal hearing demonstrated significantly higher SDS (96.7 ± 6.1%) and K-MMSE (26.4 ± 3.8) scores. In contrast, those with profound hearing loss demonstrated significantly lower SDS (26.8 ± 33.5%) and lower cognitive (K-MMSE: 23.7 ± 4.8) scores (Table 2). The prevalence of cognitive impairment increased with hearing loss severity, affecting 75.4% of individuals with mild-to-moderate hearing loss (301/399) and 84.6% with severe-to-profound hearing loss (137/162). The crude PR for cognitive impairment in the severe-to-profound group compared to the mild-to-moderate group was 1.12 (95% CI: 1.03–1.22, *p* < 0.05).

As cognitive severity increased, SRT increased and SDS declined in a stepwise manner. For example, the mean right-ear SDS was 84.4% in cognitively normal participants, decreasing to 53.6% in individuals with moderate to severe dementia. Similarly, K-MMSE scores declined progressively from 27.7 ± 2.5 in normal individuals to 25.7 ± 3.4 in individuals with MCI, 21.6 ± 4.0 in individuals with mild dementia, and 16.8 ± 5.2 in individuals with moderate to severe dementia (Table 3).

**Figure 2:**
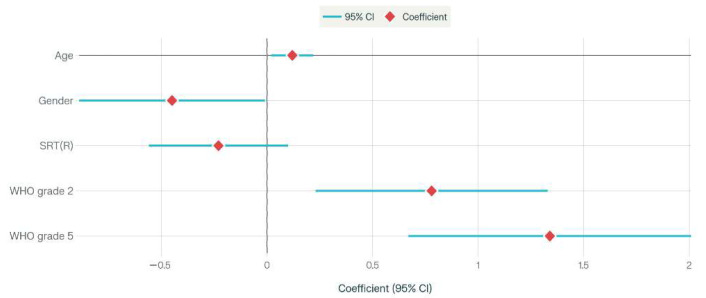
Forest plot showing adjusted coefficients (with 95% confidence intervals) for significant predictors of cognitive impairment in older adults. Negative coefficients indicate protective factors, while positive coefficients indicate risk factors. Outcomes are based on multivariate regression results.

Multivariate logistic regression identified several significant predictors of cognitive impairment among older adults with hearing loss. As illustrated in the forest plot (Figure 2), older age was significantly associated with increased odds of cognitive impairment (OR = 1.06; 95% CI: 1.03–1.09; *p* < 0.001). Male sex was associated with lower odds (OR = 0.51; 95% CI: 0.35–0.74; *p* < 0.001). A higher speech recognition threshold in the right ear (SRT(R); OR = 1.02, 95% CI: 1.01–1.04, *p* = 0.001) was also a significant predictor. Both mild hearing loss (WHO grade 2) and profound hearing loss (WHO grade 5) were significantly associated with cognitive impairment (OR = 0.57; 95% CI: 0.35–0.94; *p* = 0.021 and OR = 0.19; 95% CI: 0.05–0.72; *p* = 0.012, respectively). In contrast, SDS and WHO grades 3–4 did not reach statistical significance. The forest plot provides a visual summary of these findings, highlighting which patient characteristics most strongly predict cognitive deterioration, thereby supporting the clinical utility of integrating word recognition and cognitive testing to identify individuals at high risk of cognitive decline (Figure 2).

## 4. Discussion

This study investigated the association between hearing ability and cognitive function in older adults using a large, hospital-based dataset. Greater hearing loss, particularly when classified according to WHO criteria, was significantly associated with lower cognitive performance. This relationship was evident in both global cognitive screening using K-MMSE and in domain-specific deficits identified through the Seoul Neuropsychological Screening Battery (SNSB).

Domain-specific analysis of the SNSB provided further insight into the cognitive effects of hearing loss. Participants with lower speech recognition scores exhibited more pronounced impairments in memory and executive function, while attention and language were less affected [21,22]. These findings are consistent with previous research demonstrating that hearing loss disproportionately impacts cognitive domains dependent on auditory-verbal processing and working memory [23,24]. Mechanistically, auditory degradation impacts neural systems involved in working memory, cognitive flexibility, and goal-oriented processing—functions essential for verbal communication in complex environments [25]. This pattern is consistent with both the sensory deprivation and cognitive resource hypotheses.

The findings also support the cognitive load hypothesis, which proposes that increased listening effort under hearing-impaired conditions draws cognitive resources away from higher-order tasks such as memory and reasoning [23]. Such cognitive resource reallocation may impair daily activities, including medication adherence, financial planning, and social engagement. Recognizing these interrelationships underscores the importance of early hearing evaluation and cognitive screening. Early intervention—through hearing rehabilitation, cognitive training, or social support—may mitigate downstream effects on cognitive health and everyday functioning [6,26].

Although cognitive function was categorized into global stages—normal cognition, MCI, mild dementia, and moderate–severe dementia—this study did not differentiate specific cognitive subtypes. However, existing evidence suggests that the etiology of cognitive decline may influence its association with hearing loss [9]. For example, Alzheimer’s disease, marked by hippocampal atrophy, may be more strongly linked to memory deficits and impaired top-down auditory processing [27,28]. In contrast, vascular cognitive impairment, associated with microvascular pathology, may concurrently affect both cerebral and cochlear perfusion [29]. These mechanistic differences indicate the need for future studies using biomarkers or neuroimaging to clarify the diverse pathways linking hearing loss and cognitive dysfunction. Identifying whether specific cognitive profiles (e.g., amnestic versus dysexecutive) are more closely associated with auditory deficits could refine cognitive screening and auditory intervention strategies.

The current findings align with prior longitudinal and neuroimaging research [1,30,31]. Lin et al. reported a 30–40% increase in cognitive decline among individuals with hearing loss [1]. Deal et al. found that hearing impairment correlated with reduced brain volume in regions involved in memory and language processing [32]. Unlike studies relying solely on pure-tone thresholds, our analysis incorporated SDS, providing a more functionally relevant measure of everyday communication ability [33].

Participants with profound hearing loss exhibited significantly lower SDS and cognitive scores than those with normal hearing. This trend was consistent bilaterally, suggesting that reduced auditory input may either contribute to or reflect broader neurodegenerative changes. Logistic regression confirmed that profound hearing loss (WHO_both5) independently predicted cognitive impairment, even after adjusting for age, sex, and other auditory variables. Including the prevalence ratio in our analysis revealed that severe-to-profound hearing loss was associated with a 12% higher prevalence of cognitive impairment compared to mild-to-moderate hearing loss. This finding supports that the severity of hearing loss exerts a direct and clinically meaningful effect on cognitive health in older adults. These results support the sensory deprivation hypothesis, which posits that reduced auditory input underactivates cortical areas involved in attention, memory, and executive function [15,34].

Interestingly, only mild (WHO grade 2) and profound (WHO grade 5) hearing loss were significantly associated with cognitive impairment. Moderate and severe grades (WHO 3–4) showed no such association. This non-linear pattern may reflect varying vulnerability to cognitive decline across hearing loss stages. Mild hearing loss, often undiagnosed and untreated, may elevate listening effort and cognitive load during communication, potentially taxing attentional and working memory resources. Conversely, profound loss may signify long-standing sensory deprivation, which may accelerate neurodegeneration through reduced cortical stimulation and social withdrawal [35,36]. The absence of significant findings in the moderate and severe groups may reflect smaller sample sizes, hearing aid use, cognitive reserve, or transitional impairment not fully captured in this cross-sectional analysis.

The forest plot provides a concise and interpretable summary of predictors of cognitive impairment in older adults with hearing loss. By focusing on significant factors—including age, hearing loss severity, and speech discrimination ability—the analysis directly informs clinical risk stratification and targeted interventions through speech audiometry and cognitive screening tools. These results underscore the practical value of combined audiological and neuropsychological assessment, consistent with the study’s primary aim.

Interestingly, the non-linear association observed—where only mild and profound hearing loss demonstrated significant links to cognitive impairment—suggests that underlying mechanisms extend beyond a simple dose–response relationship [37]. Mild hearing loss may increase cognitive load and listening effort, resulting in early neural strain and functional reorganization. In contrast, profound hearing loss could drive sensory deprivation, hippocampal vulnerability, and accelerated neurodegeneration [4,38]. For moderate or severe hearing loss, compensatory strategies such as hearing aid use, behavioral adaptation, or plasticity within attentional and frontal networks may partially mitigate cognitive risks [39]. Additionally, the “common cause” hypothesis posits shared neurodegenerative or vascular processes could simultaneously affect both hearing and cognition, while psychosocial mediators such as depression, loneliness, and reduced linguistic stimulation may play differential roles across severity strata [40,41]. Considering these mechanisms together provides a more robust framework for understanding the observed unique non-linear pattern and highlights areas for future mechanistic research.

SDS declined progressively with greater cognitive impairment, suggesting that central auditory processing may be sensitive to cognitive decline [42,43]. Although SDS is primarily a measure of peripheral auditory function, reduced scores in cognitively impaired individuals may reflect deficits in top-down processing or diminished cognitive control during auditory tasks. Cognitive domains such as attention, processing speed, and working memory directly influence SDS performance. Therefore, the observed SDS decline may result from both auditory and cognitive limitations. However, stratified analysis revealed no significant interaction between SDS and cognitive group, implying that SDS declines uniformly across cognitive statuses. One study using EEG found that neural encoding of speech cues remained intact in both cognitively impaired and cognitively intact older adults, suggesting that core neural speech processing mechanisms are resilient [44]. Similarly, the Health ABC study reported that hearing impairment independently predicted cognitive decline, while cognitive status did not affect the trajectory of SDS reduction. Participants with moderate–severe hearing loss had an increased dementia risk (HR: 1.55), yet SDS declined similarly across cognitive groups after adjustment for age and cardiovascular factors [32].

Beyond its cross-sectional association with cognition, SDS may serve as a predictive marker for future cognitive decline [1]. Because SDS performance was linked to multiple domains, including memory and executive function, it may reflect early neural vulnerability before clinical dementia onset [9]. Longitudinal studies have shown that reduced speech recognition, even with near-normal pure-tone thresholds, predicts faster cognitive deterioration [45]. Therefore, incorporating SDS into dementia risk models may improve early detection, especially in resource-constrained settings. SDS should be viewed not merely as an auditory acuity measure but as a composite indicator of auditory cognition. Accurate word recognition demands central auditory integration, sustained attention, working memory, and linguistic comprehension—cognitive abilities that decline with age and neurodegeneration [46,47]. Framing SDS as a cognitive–auditory marker may clarify its association with impairments in cognitively demanding domains [33,48].

Age was a strong, consistent predictor of both hearing and cognitive decline, supporting the notion that these are parallel neurodegenerative processes in aging [38,49]. Additionally, male sex was linked to a higher risk of cognitive impairment [50]. This may be due to differences in occupational noise exposure, healthcare utilization, or psychosocial variables, though further investigation is needed.

Educational level did not differ significantly between cognitive groups but was slightly higher among those with normal cognition. This may reflect the protective role of cognitive reserve, allowing continued function despite underlying neuropathology [51,52]. Although this effect was not statistically significant, it merits further exploration in longitudinal analyses.

These findings underscore the clinical relevance of including audiological assessments in cognitive evaluations of older adults. The consistent association between hearing loss—especially reduced speech recognition—and cognitive impairment suggests that hearing loss may be both a marker and potential contributor to cognitive decline. Interventions such as hearing aids, assistive devices, and auditory training should be explored for their potential to slow cognitive deterioration.

This study has several limitations. Cognitive impairment may have affected audiometric performance, particularly in speech discrimination tasks requiring attention, short-term memory, and linguistic processing. Therefore, some SDS decline may result from cognitive, not purely auditory, deficits. The cross-sectional design limits causal inference, and residual confounding from factors such as depression, social isolation, or socioeconomic status cannot be excluded. Additionally, the relatively high mean age of participants may have contributed to lower speech discrimination scores due to age-related cognitive slowing, independent of auditory processing deficits. Therefore, caution should be exercised when generalizing these findings to younger adults. Hearing loss may have influenced auditory-based cognitive assessments, potentially underestimating performance in patients with uncorrected hearing loss. Nonetheless, the study’s large sample size and detailed neuropsychological assessments enhance the validity of the findings. Potential confounders such as age, sex, and education were adjusted in the statistical models. However, residual confounding from variables such as depression, social isolation, or uncorrected hearing loss may persist. Furthermore, the cross-sectional design of this study precludes direct inference of causality between hearing loss and cognitive impairment. The observed associations may reflect shared neurodegenerative mechanisms, reverse causality, or confounding by psychosocial factors such as reduced social engagement and depression. Although we adjusted for major covariates such as age, sex, and education, unmeasured confounders may persist, and disentangling these alternative explanations remains challenging. Further longitudinal studies are needed to clarify causal pathways and temporal relationships.

In conclusion, this study demonstrated that poorer SDS and greater hearing loss severity (WHO classification) were significantly associated with cognitive impairment in older adults. Both mild and profound hearing loss were independently related to lower cognitive performance, particularly in memory and executive function, as assessed by K-MMSE and SNSB. These findings suggest that speech discrimination reflects not only peripheral auditory sensitivity but also central auditory–cognitive integration. Incorporating speech audiometry into routine cognitive screening may help identify individuals at risk for cognitive decline and support early, dual-domain interventions for hearing and cognitive health.

## Figures and Tables

**Table 1:** General characteristics of the study population.

	Total Patients (n = 801)
Age	77.10 ± 9.77
Sex (M:F)	313 (39.1%)/488 (60.9%)
SRT (right)	39.69 ± 24.91
WHO classification (right)	
Normal	303 (37.8%)
Mild	240 (30.0%)
Moderate	149 (18.6%)
Severe	22 (2.7%)
Profound	87 (10.9%)
SRT (left)	41.44 ± 26.71
WHO classification (left)	
Normal	296 (37.0%)
Mild	228 (28.5%)
Moderate	149 (18.6%)
Severe	19 (2.4%)
Profound	109 (13.6%)
SDS (right)	73.35 ± 29.92
SDS (left)	71.23 ± 31.79
Education (years)	10.74 ± 5.24
K-MMSE	25.14 ± 4.33
CDR	
0	70 (8.7%)
0.5	632 (78.9%)
1.0	72 (9.0%)
2.0	26 (3.2%)
3.0	1 (0.1%)
Dementia	
Normal	205 (25.6%)
MCI	438 (54.7%)
Mild dementia	115 (14.4%)
Moderate to severe	43 (5.3%)

Abbreviations: SRT, speech recognition threshold; SDS, speech discrimination score; K-MMSE, Korean Mini-Mental State Examination; CDR, Clinical Dementia Rating; MCI, mild cognitive impairment. Descriptive statistics only.

**Table 2:** Participant characteristics according to hearing loss severity based on the WHO classification (worse ear).

	Normal (n = 240, 30.0%)	Mild (n = 234, 29.2%)	Moderate (n = 165, 20.6%)	Severe (n = 29, 3.6%)	Profound (n = 133, 16.6%)	*p*-Value
Age	71.56 ± 9.76	77.39 ± 7.65	79.60 ± 9.02	83.41 ± 6.64	82.11 ± 9.74	<0.001
Sex (M:F)	83:157 (34.6%:65.4%)	78:156 (33.3%:66.7%)	61:104 (37.0%:63.0%)	19:10 (65.5%:34.5%)	72:61 (54.1%:45.9%)	<0.001
SRT_R	18.77 ± 4.65	32.49 ± 6.31	45.07 ± 10.07	60.34 ± 13.22	78.94 ± 30.31	<0.001
SRT_L	18.63 ± 4.60	32.29 ± 6.91	46.24 ± 9.06	60.52 ± 15.60	88.56 ± 24.67	<0.001
SDS_R	96.71 ± 6.10	82.90 ± 13.20	66.24 ± 17.53	57.07 ± 20.03	26.80 ± 33.54	<0.001
SDS_L	95.99 ± 10.20	83.15 ± 11.74	64.43 ± 17.35	56.41 ± 22.77	17.24 ± 28.26	<0.001
Education (years)	11.71 ± 4.88	10.31 ± 5.44	10.04 ± 5.37	11.24 ± 5.42	10.48 ± 5.12	0.010
K-MMSE	26.43 ± 3.78	25.23 ± 4.27	24.51 ± 4.25	23.86 ± 4.64	23.73 ± 4.77	<0.001
CDR						0.002
0	34 (14.2%)	17 (7.3%)	9 (5.5%)	0 (0.0%)	10 (7.5%)	
0.5	188 (78.3%)	190 (81.2%)	134 (81.2%)	26 (89.7%)	94 (70.7%)	
1.0	14 (5.8%)	18 (7.7%)	18 (10.9%)	1 (3.4%)	21 (15.8%)	
2.0	3 (1.3%)	9 (3.8%)	4 (2.4%)	2 (6.9%)	8 (6.0%)	
3.0	1 (0.4%)	0 (0.0%)	0 (0.0%)	0 (0.0%)	0 (0.0%)	
Cognition status						<0.001
Normal	82 (34.2%)	69 (29.5%)	29 (17.6%)	2 (17.3%)	23 (25.6%)	
MCI	125 (52.1%)	120 (51.3%)	106 (64.2%)	20 (50.4%)	67 (54.7%)	
Mild dementia	27 (11.3%)	34 (14.5%)	20 (12.1%)	4 (22.6%)	30 (14.4%)	
Moderate to severe	6 (2.5%)	11 (4.7%)	10 (6.0%)	3 (9.1%)	13 (5.3%)	

WHO, World Health Organization; SRT_R/L, speech recognition threshold (right/left ear); SDS_R/L, speech discrimination score (right/left ear); K-MMSE, Korean Mini-Mental State Examination; CDR, Clinical Dementia Rating; MCI, mild cognitive impairment. Group comparisons by one-way ANOVA (continuous variables) and chi-square test (categorical variables). Group Comparisons by Cognitive Function.

**Table 3:** Participant characteristics according to cognitive function status.

	Normal (n = 205, 25.6%)	MCI (n = 438, 54.7%)	Mild (n = 115, 14.4%)	Moderate to Severe (n = 43, 5.3%)	*p*-Value
Age	71.98 ± 9.93	77.69 ± 9.16	81.23 ± 8.05	84.49 ± 7.50	<0.001
Sex (M:F)	52:153 (25.4%:74.6%)	187:251 (42.7%:57.3%)	56:59 (48.7%:51.3%)	18:25 (41.9%:58.1%)	<0.001
SRT_R	30.68 ± 20.88	41.06 ± 24.12	44.96 ± 27.16	54.65 ± 30.46	<0.001
SRT_L	34.68 ± 24.43	41.26 ± 25.49	50.35 ± 30.58	51.58 ± 29.15	<0.001
WHO classification					<0.001
Normal	82 (40.0%)	125 (28.5%)	27 (23.5%)	6 (14.0%)	
Mild	69 (33.7%)	120 (27.4%)	34 (29.6%)	11 (25.6%)	
Moderate	29 (14.1%)	106 (24.2%)	20 (17.4%)	0 (23.3%)	
Severe	2 (1.0%)	20 (4.6%)	4 (3.5%)	3 (7.0%)	
Profound	23 (11.2%)	67 (15.3%)	30 (26.1%)	13 (30.2%)	
SDS_R	84.44 ± 24.54	71.91 ± 29.13	66.50 ± 32.18	53.58 ± 36.55	<0.001
SDS_L	81.14 ± 28.24	70.64 ± 30.46	61.32 ± 36.30	56.47 ± 34.12	<0.001
Education (years)	10.89 ± 5.38	10.70 ± 5.03	10.77 ± 5.70	10.34 ± 5.54	0.929
K-MMSE	27.67 ± 2.52	25.70 ± 3.37	21.64 ± 3.96	16.81 ± 5.19	<0.001
CDR					<0.001
0	52 (25.4%)	18 (4.1%)	0 (0.0%)	0 (0.0%)	
0.5	152 (74.1%)	413 (94.3%)	67 (58.3%)	0 (0.0%)	
1.0	1 (0.5%)	5 (1.1%)	46 (40.0%)	20 (46.5%)	
2.0	0 (0.0%)	2 (0.5%)	2 (1.7%)	22 (51.2%)	
3.0	0 (0.0%)	0 (0.0%)	0 (0.0%)	1 (2.3%)	

SRT_R/L, speech recognition threshold (right/left ear); SDS_R/L, speech discrimination score (right/left ear); WHO, WHO hearing classification based on worse ear; K-MMSE, Korean Mini-Mental State Examination; CDR, Clinical Dementia Rating; MCI, mild cognitive impairment. Group comparisons by one-way ANOVA (continuous variables) and chi-square test (categorical variables).

## Data Availability

The datasets generated and analyzed during the current study are not publicly available due to institutional policy, but are available from the corresponding author upon reasonable request.
